# Lens-Induced Glaucoma: An Audit of Cataract Cases in Hospital Sultanah Nur Zahirah, Kuala Terengganu (HSNZKT)

**DOI:** 10.7759/cureus.22875

**Published:** 2022-03-05

**Authors:** Aini Mohd Azmi, Nor Anita Che Omar, Wan Haslina Wan Abdul Halim

**Affiliations:** 1 Ophthalmology, Hospital Canselor Tuanku Muhriz UKM (Universiti Kebangsaan Malaysia), Kuala Lumpur, MYS; 2 Ophthalmology, Hospital Sultanah Nur Zahirah, Kuala Terengganu, MYS

**Keywords:** ned, cataract, lens-induced glaucoma, phacomorphic, phacolytic

## Abstract

Introduction: Cataract is the main cause of preventable blindness worldwide and as such, it is important to identify these patients early before complications develop. Cataracts may progress to become intumescent (phacomorphic) or hypermature (phacolytic). This can lead to lens-induced glaucoma, which may subsequently cause permanent optic nerve damage.

Objective: To report on lens-induced glaucoma (LIG) of cataract cases treated in Hospital Sultanah Nur Zahirah, Kuala Terengganu (HSNZKT), Malaysia.

Method: National Eye Database (Malaysia) data from 2011 till 2017 were collected and analysed.

Results: A total of 81 (1.08%) cases of lens-induced glaucoma were calculated from 7468 cataract cases treated during the seven years of the study period. The number of cases showed an increasing trend with 0.78% in 2011 to 1.26% in 2017. Most of the patients were in the age group of 70-79 years (44.30%), followed by 60-69 years (34.18%), 50-59 years (11.39%), 80-89 years (7.59%), 40-49 years, and 90-99 years (1.27%). Most of the cases are female (57%). The majority of them (79.75%) underwent cataract operation for the first eye.

Conclusion: Overall, this study was able to highlight the significant association between the incidence of LIG and increasing age as well as surgery series (either first or second eye). Therefore, more outreach programs should be conducted in the future to enable younger elderly patients from all areas to receive treatment. More online education and talk series can be organized to increase the community’s awareness and acceptance of cataract operations.

## Introduction

Cataract is one of the leading causes of blindness and visual impairment in Malaysia [1]. Worldwide, it is the number one cause of preventable blindness [2]. It is a painless, progressive opacification of the crystalline lens that is treatable by surgery (lens extraction) [2]. If a cataract is left untreated, it can progress to become intumescent or hyper mature, leading to lens-induced glaucoma (LIG) [3,4]. Among the types of LIG are phacomorphic and phacolytic glaucomas. These two lens conditions are vision-threatening complications of cataracts, which may be prevented by early treatment of the cataract [4].

This study was presented as a poster at the 8th Conjoint Ophthalmology Scientific Conference 2018, Malaysia, September 15-16, 2018 [5]. It won the Best Poster award.

## Materials and methods

This study aims to measure the prevalence of LIG among patients who had undergone surgical treatment for the cataract as well as to describe their sociodemographic data. This is a retrospective study whereby data was collected from the National Eye Database (NED), Malaysia, from January 1, 2011, to December 31, 2017. A total of 7468 operations were done during this period and a total of 81 patients who had LIG at the time of operation were included in this study. The patients’ age, gender, type of LIG, and whether the surgery was for the first or second eye were recorded.

## Results

A total of 81 (1.08%) cases of LIG were identified from 7468 cataract cases treated during the seven years study period. The number of LIG cases showed an increasing trend with six cases (0.78%) in 2011 to 20 cases (1.26%) in 2017 (Table 1). Of these, 65.82% were phacomorphic while 34.18% were phacolytic. Most patients were in the age group of 70-79 years (44.30%), followed by 60-69 years (34.18%), 50-59 years (11.39%), 80-89 years (7.59%), and finally 40-49 years and 90- 99 years old at 1.27% each (Table 2). More female patients (56.96%) presented with this problem. The majority (79.75%) underwent cataract operation for the first eye (Table 2).

**Table 1:** Descriptive Data of the Lens-Induced Glaucoma Cases LIG: Lens-Induced Glaucoma; N: Number; %: Percentage

			N	%
Age categories (years)		40-49	1	1.27
		50-59	9	11.39
		60-69	27	34.18
		70-79	35	44.30
		80-89	6	7.59
		90-99	1	1.27
Gender		Male	42	43.00
		Female	45	57.00
Year of cases		2011	6	0.78
		2012	5	0.65
		2013	7	0.69
		2014	9	0.95
		2015	15	1.48
		2016	17	1.37
		2017	20	1.26
Race		Malay	79	97.5
		Chinese	2	2.50
		Others	0	0
Operation series		First eye	63	80.00
		Second eye	16	20.00
Types of LIG		Phacomorphic	52	64.20
		Phacolytic	29	35.80

**Table 2:** Significant Variables Associated with Lens-Induced Glaucoma a Chi-Square test b Kruskal-Wallis test * significant p-value < 0.05

Variables			Number	Chi-Square / Kruskal-Wallis Value	p-value
Gender	Male	36		0.671^a^	0.436
	Female	45			
Year of cases	2011	6		7.010^a^	0.320
	2012	5			
	2013	7			
	2014	9			
	2015	15			
	2016	17			
	2017	20			
Race	Malay	79		1.375^a^	0.967
	Chinese	2			
	India	0			
	Others	0			
Operation series	First eye		65	4.575^a^	0.039*
	Second eye		16		
Age (years)	Mean 63.93			13.472^b^	0.000*

## Discussion

Cataract is one of the commonest eye diseases referred to the ophthalmology team for surgical intervention [6]. It is multifactorial but aging is the most common cause [7]. In this study, it is highlighted that cataract can progress to become intumescent (phacomorphic) or hypermature (phacolytic) leading to LIG, which can subsequently cause permanent optic nerve damage. Over the years, there has been an increase in the number of cases of LIG in HSNZKT. This increasing incidence may be contributed by the increase in the elderly population because of improvement in healthcare [4]. It is estimated that the prevalence of cataract among the elderly population in Malaysia is up to 30% in which more cataract-related complications may arise, including LIG [8].

In this study, more patients presented with phacomorphic lens than phacolytic lens, which is similar to results from other studies that has been done (range from 57.5% to 73.7%) with incidence per year of about 37 to 40 cases [3,4,9]. In HSNZKT, there were more female patients who presented with LIG and similar results had been obtained in other studies [3,4,9]. This may be due to higher prevalence of cataract among them [4,9]. In Malaysian National Eye Survey II, it was found that females are less likely to seek treatment for cataract surgery compared to males [1] and, therefore, may present late with their cataract problem. This is most likely due to the lack of emphasis placed on them for seeking treatment. This shows that gender bias may put females at higher risk due to lower priority given to them in some communities to seek treatment [1,3,9]. However, there is no significant difference in terms of gender associated with LIG in this study (Table 2).

As shown in this study, incidence of LIG is highest in those in the age group of 70-79 years followed by the age group of 60-69 years. This finding is similar to other studies, which showed the highest prevalence in those aged 60-80 years [3,4]. This study also proved that increasing age is significantly associated with LIG as shown in Table 2 (p-value 0.00). Both types of LIG are associated with late presentations [4]; therefore, those in the older age groups who were dependent on others to seek treatment, predominates.

Most of the patients with LIG underwent operations for the first eye, which is statistically significant in our study (p-value 0.039) as reflected in Table 2. This finding is comparable to other surveys in which LIG is detected on first consultation with the ophthalmologist [10]. It is best to counsel these patients about timely surgery in the second eye [8] to prevent similar complications. Efforts should continue to be done to increase accessibility to surgical care and to manage other contributing factors such as financial and social factors that prevent patients from going for cataract operation [1].

## Conclusions

Overall, this study was able to highlight the significant association between the incidence of LIG and increasing age as well as surgery series (either first or second eye). Therefore, more outreach programs should be conducted in the future to enable younger elderly patients from all areas to receive treatment. More online education and talk series can be organized to increase the community’s awareness and acceptance of cataract operations.
